# Effects of Pine Pollen Polysaccharides and Sulfated Polysaccharides on Ulcerative Colitis in Mice by Regulating Th17/Treg

**DOI:** 10.3390/foods13193183

**Published:** 2024-10-07

**Authors:** Zhanjiang Wang, Zhenxiang Li, Hanyue Wang, Qiu Wu, Yue Geng

**Affiliations:** Key Laboratory of Food Nutrition and Safety of SDNU, College of Life Science, Shandong Normal University, Jinan 250358, China; 2018020835@stu.sdnu.edu.cn (Z.W.); lzx17853136900@163.com (Z.L.); eunicewan@163.com (H.W.); wuqiu@sdnu.edu.cn (Q.W.)

**Keywords:** pine pollen polysaccharide, ulcerative colitis, Treg cells, Th17 cells, intestinal flora

## Abstract

This study was to investigate the effects of the polysaccharides (PPM60−III) and sulfated polysaccharides (SPPM60−III) of pine pollen on the Th17/Treg balance, inflammatory cytokines, intestinal microbiota, and metabolite distribution in 3% DSS drinking water-induced UC mice. First of all, the physiological results showed that PPM60−III and SPPM60−III could alleviate UC, which was shown by the reduction in liver Treg cells, the rebalance of Th17/Treg, and the modulation of inflammatory cytokines. In addition, the 16S rRNA results showed that PPM60−III and SPPM60−III could decrease *Beijerinck* and *Bifidobacterium*, and increase *Akkermansia*, *Escherichia coli*, and *Fidobacteria*. Finally, the metabonomics results showed that PPM60−III and SPPM60−III also restored purine and glycerolipid metabolism, up-regulated nicotinate and nicotinamide metabolism and caffeine metabolism to inhibit inflammation. In conclusion, PPM60−III and SPPM60−III could inhibit UC by regulating gut bacteria composition and metabolite distribution; SPPM60−III showed better anti-colitis activity.

## 1. Introduction

Ulcerative colitis (UC) is an inflammatory bowel disease that occurs in the colon tissue and submucosa. Its clinical manifestations are abdominal pain, diarrhea, mucous purulent, and bloody stool. The ulcer surface of the colon slowly extends to the entire colon [1]. The incidence of UC is increasing worldwide. In the United States and Europe, nearly 1 million people are affected by this disease, and globally it is even more [2]. The development process of chronic UC is inflammation–hyperplasia–canceration–colorectal cancer [3]. The etiology of UC is unknown. Individual genetic susceptibility, environmental factors, intestinal microflora, and immune response are all factors of UC, and all these factors work together [4]. Dysfunction of the immune pathway can lead to abnormal intestinal inflammatory response [5]. UC is an intestinal inflammatory response driven by Th2 response and involving Th17 cells [6]. Excessive inflammation is one of the most typical characteristics of UC. An imbalance in the NF-κB signal pathway leads to excessive inflammation [7]. The main manifestation of UC is the imbalance of Th17/Treg cells [8]. During the onset of UC, Th17 increases while Treg decreases, and the intestinal crypt structure changes [9,10]. Th17 cells are a subset of T cells that cause the secretion of IL-17 pro-inflammatory cytokines [11]. IL-17, as a key cytokine, has six kinds of receptor proteins including IL-17A to IL-17F, which can activate the NF-κB pathway, and cause inflammation and tissue infiltration [12,13]. The NF-κB pathway can cause the release of IL-1β and TNF-α inflammatory factors. IL-17 promotes IL-6 and TNF-α secretion, leading to inflammatory cells infiltrating the intestinal mucosa, thereby causing damage to intestinal tissue [14].

Treg cells produce IL-10 and TGF-β through a contact-dependent mechanism [15]. After binding to its receptor, IL-10 activates Janus kinase 1 and tyrosine kinase 2, phosphorylates the downstream target genes, and inhibits inflammation [16]. IL-10 inhibits the production of effector T cells and the release of pro-inflammatory cytokines and plays an immunosuppressive role [17]. TGF-β is an inflammatory marker of UC [18]. It affects Treg and Th17 cells [19]. Treg cells, expressing CD 39 and CD 73, have a variety of immunosuppressive functions. They can disrupt the metabolism of effector T cells, promote the secretion of IL-10 and TGF-β, and inhibit the function of Th17 cells [20]. “Kuijie enema” is a traditional Chinese medicine prescription for clinical treatment of UC [21]. It has been found that it can regulate the immune balance of Th17/Treg, increase the number of Treg cells, up-regulate the level of IL-10, reduce the number of Th17 cells, down-regulate IL-17A and TNF-α, and improve UC [22]. According to the clinical investigation, when the Th17/Treg was abnormal in UC patients, the serum levels of pro-inflammatory factors IL-6, IL-17A, and IL-17F increased sharply [23]. The contents of Treg-related anti-inflammatory factors such as TGF-β and IL-10 decreased [24]. The study of Han nationality patients found that when Th17/Treg was out of balance, the content of pro-inflammatory factor IL-17 in serum increased, while Th17 cells increased, anti-inflammatory factor TGF-β1 decreased, and Treg cells decreased [25]. All in all, UC induced by high DSS ingestion is a very serious problem, and therefore, it is necessary to further develop safe and active ingredients that can prevent such damage.

Yunnan pine pollen is the male gametophyte of *Pinus yunnanensis*, which has a long history of medicinal use in China. The *Compendium of Materia Medica* records both its sweet taste and important functions such as returning blood back to the liver and spleen meridian, moistening the heart and lung, tonifying qi, removing wind, stopping bleeding, and so on [26]. Our previous work showed that pine pollen polysaccharides (PPM60−III) have certainly immune activity [27]. But natural polysaccharides have relatively weak biological activity, limiting their potential applications. In order to maximize their effectiveness, researchers have explored various modifications of polysaccharides and found that sulfation is one of the important chemical modifications that enhances their bioactivity. We have sulfated the Yunnan pine pollen polysaccharides (SPPM60−III), analyzed the structure, and found that the activity significantly increases in areas such as antioxidant and anti-tumor properties [27].

With this in mind, animal experiments were designed. The mice were fed with 3% DSS drinking water; PPM60−III and SPPM60−III were prepared by using our previous methods [27] and they were used to evaluate the protective effect of PPM60−III and SPPM60−III on DSS-induced UC by testing the number of Th17 cells, Th17/Treg balance, the levels of inflammatory cytokines (IL-1β, IL-6, TNF-α, IL-10, IL-17A, and TGF-β), the metabolite distribution of serum, and the composition of intestinal flora. These findings may help us better understand the protective effect of PPM60−III and SPPM60−III on UC mice, elucidate structure–activity relationships of them, and are of great significance for further development of Yunnan pine pollen.

## 2. Materials and Methods

### 2.1. Materials and Reagents

Broken masson pine (*Pinus yunnanensis* Franch, broken rate > 95%) pollen were provided by Yantai New Era Health Industry Co., Ltd. (Yantai, China). Dextran sulfate sodium salt (DSS, 36,000–50,000 Da) was purchased from MP Biomedicals (Santa Ana, CA, USA). Hematoxylin and eosin (H&E) were obtained from Wuhan Servicebio Biological Co., Ltd. (Wuhan, China).

Mouse IL-1β, IL-6, IL-17, TNF-α, IL-10, and TGF-β ELISA kits; mouse regulatory T cell staining kit 70-KTH217-25; and mouse Th17 staining kit for flow cytometry were purchased from MultiSciences (Lianke) Biotech Co., Ltd. (Hangzhou, China). D_2_O (deuterium oxide, 99.9 atom % D, 0.05% TSP) was bought from Sigma-Aldrich (Saint Louis, MO, USA). NORELL NMR tubes (Morganton, NC, USA) and Corning cryopreservation tubes (Corning, NY, USA) were used.

### 2.2. Preparation of PPM60−III and SPPM60−III

Broken masson pine pollen polysaccharides were extracted by using a water extract–alcohol precipitation method. Polysaccharide (PPM60) was extracted by hot water, and precipitated by 60% ethanol. Proteins were removed by using the trichloroacetic acid precipitation method. PPM60−III was separated and purified from PPM60 by Sephacryl^TM^ S-400HR (GE-Healthcare Bio Sciences Ab, Marlborough, MA, USA). The content of polysaccharides was determined by the phenol sulfuric acid method, and the OD value was measured at 490 nm. The curve was drawn by taking the number of test tubes collected as the abscissa and the OD value as the ordinate [28]. The calculation formula of the esterification degree was DS = (162 × S)/(32−102 × S). The absorbance value was taken as the abscissa and the sulfate concentration as the ordinate to draw the sulfate standard curve. Similarly, the absorbance of the sample was measured, the absorbance value substituted into the standard curve, and the concentration of sulfate calculated.

Sulfated polysaccharide III (SPPM60−III), with a degree of substitution of 1.21, was made by using our previous method: chlorosulfonic acid–pyridine [27]. The specific process is as follows. A mortar, pre-cooled in a refrigerator, was brought out and placed on ice in the fume hood for the following experiment. A volume of 2 mL of pyridine was taken and added into the mortar. Then, the pipette gun was used to slowly add 2 mL of chlorosulfonic acid along the mortar wall; the process should be rapid and continuous, and stirring is needed. The ice bath was removed as soon as white solids appeared in the mortar and ground until a yellowish-white thick liquid was formed. The sulfonation reagent was obtained when the reagent was melted. A mass of 100 mg of pinus yunnanensis pollen polysaccharide was dissolved in 6 mL of anhydrous formamide in a 50 mL centrifuge tube. Then, the sulfonation reagent was slowly added, while constantly stirring to mix it evenly, and then, put in a 45 °C water bath. The reaction proceeded for 4 h, with oscillatory mixing every 30 min. After the reaction was completed, the mixed solution was adjusted to a neutral pH with 2.5 mol/L sodium hydroxide, and deammonification was carried out. The resulting solution was then transferred to a dialysis bag for dialysis purification; first, with tap water for 4 days, and then, with deionized water for 2 days. When the color of the solution in the dialysis bag changed from yellow to colorless, the liquid was removed from the dialysis bag and spun. Finally, the concentrated liquid was freeze-dried, and the resulting solid was weighed and named SPM60.

Polysaccharide powder was dried prior to tableting with KBr powder. IS50 Fourier transform infrared (FT-IR) spectroscopy (Medison, WI, USA) was performed to investigate the FT-IR spectrum, and detected in the frequency range of 400–4000 cm^−1^.

### 2.3. Animal Experiment

Male C57BL/6 mice (20–24 g) were purchased from Jinan Pengyue Experimental Animal Breeding Co., Ltd. (Jinan, China). The temperature was maintained at 20–25 °C and the humidity was kept at 50–60% with a 12 h/12 h light/dark cycle. All mice had free access to standard rodent chow and water for 1 week during the acclimation. All experiments were carried out according to the Guidelines for the Care and Use of Animals, and this study was evaluated and approved by the Ethics Committee of Shandong Normal University (approval no. AEECSDNU2020008) in 10 April 2020, and the experimental procedure was carried out according to the Guideline of Experimental Animal Administration published by the State Committee of Science and Technology of the People’s Republic of China.

**Figure 1:**
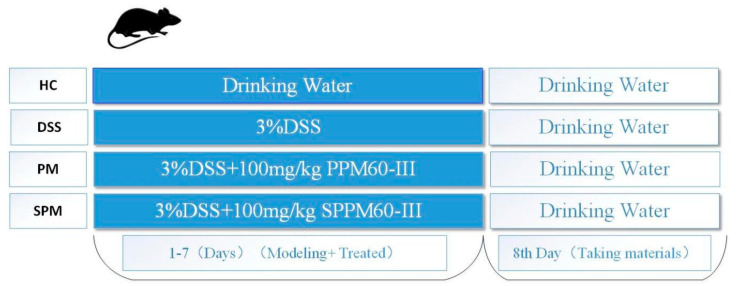
Diagram of study design.

According to the results of a preliminary experiment, it was determined that the doses of PPM60−III and SPPM60−III would be 100 mg/kg/day [29]. All animals were assigned to four groups randomly, with 10 mice in each group: (a) HC group, the control group, treated with drinking water; (b) DSS group, treated with 3% DSS for 7days; (c) PM group, administrated with 100 mg/kg·day PPM60−III for 7 days; (d) SPM group, administrated with 100 mg/kg·day SPPM60−III for 7 days. Except for the mice in the HC group, the other mice were allowed free access to 3% DSS water for 7 days (Figure 1). The mice in the PM and SPM groups were given 0.2 mL of PPM60−III and SPPM60−III, respectively. The mice in the HC and DSS groups were given the same amount of normal saline. The body weights of the mice were recorded every day. The daily diet intake and life state of the mice (such as hair condition, activity level) were recorded as well [30].

### 2.4. Assessment of DAI (Disease Activity Index)

The body weights and fecal scores of the mice were monitored daily. The DAI was determined as the sum of the body weight, the diarrheal stool score, and the bloody stool score according to the DAI scoring system [30]. The DAI scoring criteria are shown in Table 1.

### 2.5. Sample Collection

Mice were deprived of food overnight and euthanized under anesthesia by pentobarbital. Blood samples were collected and immediately centrifuged at 3000 rpm for 15 min at 4 °C to separate and collect the serum samples. Spleen, colon as well as the contents of the cecum were collected, and the weight of the liver and spleen was obtained, the length of the colon was measured.

### 2.6. Flow Cytometry

The spleens were placed in 5 mL of phosphate-buffered saline (PBS) after mice were sacrificed. A single-cell suspension of splenocytes was prepared by grinding over a nylon membrane (200 mesh) and centrifuged (500× *g*) for 5 min. The isolated cells were lysed to remove red blood cells. Standard intracellular cytokine staining was used as previously described [31]. FITC anti-mouse CD3 and PE anti-mouse CD4 were used for Th17 cell and Treg cell staining, respectively. Samples and data were collected and analyzed using LSR Fortessa (Becton, Dickinson and Company, NJ, USA) and software (CytExpert 2.0).

### 2.7. Histopathological Experiment

The distal colon was fixed in a 4% paraformaldehyde solution for at least 24 h and embedded in paraffin. Paraffin sections (5 μm thickness) were stained with hematoxylin and eosin (H&E).

### 2.8. Determination of Inflammatory Factors

The colon tissue was weighed, cut into small pieces, 9 times the volume of PBS solution (0.01 M, pH 7.4) was added, and manually ground at 4 °C to obtain a tissue homogenate for use. The levels of IL-1β, IL-6, TNF-α, IL-10, and TGF-β in the tissue homogenate were measured by ELISA according to the manufacturer’s instructions. The Spectra Max Plus microplate reader from Molecular Devices (San Jose, CA, USA) was used for detection.

### 2.9. Nuclear Magnetic Metabolomics

The serum was prepared after blood centrifugation (3000 rpm, 15 min) and stored at −80 °C. A volume of 100 μL of serum was taken and thawed to 4 °C. Each serum sample was reconstituted in 500 μL of K_2_HPO_4_-KH_2_PO_4_ buffer solution in D_2_O. D_2_O was used for field frequency lock, and TSP was used to provide the chemical shift reference (d 0.00). Subsequently, all the samples were vortexed and centrifuged at 12,000× *g* for 15 min at 4 °C to remove any insoluble components. Finally, the collected supernatants (550 μL) were transferred to 5 mm NMR tubes and analyzed by an Avance III 400 NMR spectrometer (Billerica, MA, USA) [32,33].

All NMR data were processed using the MestReNova 6.11 software from Mestrelab Research (Santiago de Compostela, Spain). The exponential window function was added to all spectra for manual baseline correction. TSP was used as a standard. Water peaks were manually removed from the data, after which the NMR spectra were normalized. Data containing integrated peaks were first exported to Excel 16.0 (Microsoft, Redmond, WA, USA), and then, to the SIMCA-P 14.1 program (Umetrics Inc., Umea, Sweden). Data analysis was performed using partial least squares–discriminant analysis.

### 2.10. Microbiota Sequencing Analysis

The collected colon content samples were sent to Beijing Novogene Biotech Co., Ltd. (Beijing, China) for testing.

Primer corresponding region: 16S V4 region primers (515F and 806R): identify bacterial diversity; 18S V4 region primers (528F and 706R): identify the diversity of eukaryotic microorganisms; ITS1 region primers (ITS5-1737F and ITS2-2043R): identification of fungal diversity.

The DNA was extracted and detected. When PCR amplification was completed, the product was purified, then tested in the prepared library, and finally, sequenced on the computer. The raw data obtained by sequencing (raw data) had a certain proportion of interference data (dirty data). First, the raw data were spliced and filtered to obtain the effective data (clean data). Then, OTU (operational taxonomic unit) clustering and species classification analysis was performed based on the valid data. According to the clustering results of the OTUs, the representative sequence of each OTU was annotated, with the corresponding species information and the abundance distribution based on the species. For the differences in community structure among grouped samples, statistical analysis methods such as *t*-test, Simper, MetaStat, LEfSe, Anosim, and MRPP were used to test the species composition and community structure of the grouped samples for significant differences.

### 2.11. Electron Microscope Analysis

To examine the ultrastructure of the colon, electron microscope analysis was carried out. Briefly, colon tissues were fixed in 2.5% glutaraldehyde (pH 7.4) and 1% osmium tetraoxide (pH 7.4). Afterwards, the samples were dehydrated by alcohol and acetone followed by embedding in araldite. Then, tissues were sliced into 60–70 nm slices and stained with uranyl acetate and lead citrate. Finally, the ultrastructure of the colon tissues was examined and photographed using an HT-7800 transmission electron microscope (Hitachi Corporation, Tokyo, Japan).

### 2.12. Statistical Analysis

All measured data are expressed as mean standard error of the mean (mean SEM). All statistical analyses were performed using SPSS version 17. All graphs were drawn with GraphPad Prism 5 (version 7.0, La Jolla, CA, USA). Significant differences were determined by one-way analysis of variance (ANOVA) followed by Tukey’s *t*-test. * *p* < 0.05, ** *p* < 0.01.

## 3. Results

### 3.1. Selection of Polysaccharide Components and Infrared Spectral Detection

**Figure 2:**
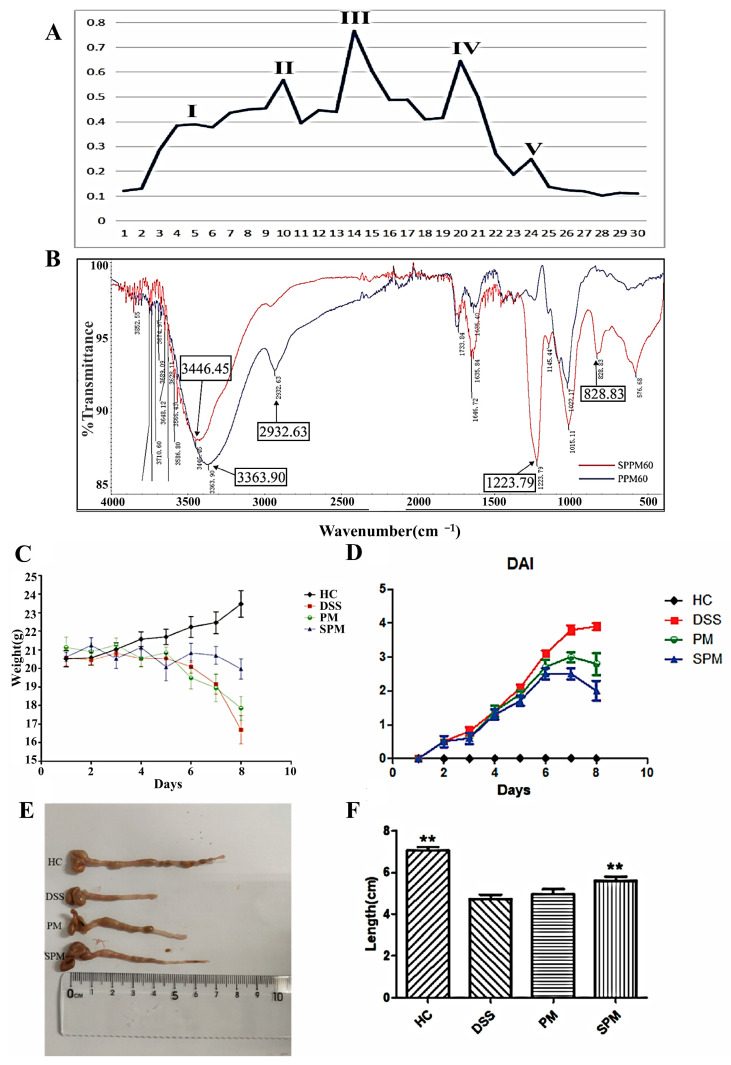
Isolation, extraction, and analysis of Yunnan pine pollen polysaccharides and improvement of UC by PPM60−III and SPPM60−III. (**A**) Chromatographic separation diagram prepared by constant pressure; (**B**) infrared spectra of PPM60−III and SPPM60−III. (**C**) Body weight change trend chart; (**D**) DAI score chart; (**E**) the appearance of colon; (**F**) the length of colon. ** *p* < 0.01.

As shown in Figure 2A, the crude polysaccharides of pine pollen were extracted by boiling water and alcohol precipitation, and the polysaccharide component PPM60 was obtained by 60% ethanol precipitation. The third component isolated from Sephacryl^TM^ S-400 by atmospheric-pressure column chromatography was named PPM60−III for follow-up experiments.

The infrared spectrum showed that PPM60−III had the characteristic absorption peaks of O-H at 3363.90 cm^−1^ and C-H at 2932.63 cm^−1^, which are the positions of the characteristic absorption peaks of polysaccharides. The characteristic absorption peak of C=O was shown at 1733.84 cm^−11^. Except for the characteristic absorption peaks of PPM60−III, SPPM60−III showed the characteristic absorption peaks of S=O at 1223.79 cm^−1^ and C-O-S at 828.83 cm^−1^. These were obviously replaced by sulfate at the characteristic absorption peak of O-H, which indicated that sulfated polysaccharides had been prepared successfully.

### 3.2. Construction and Symptom Improvement in UC Mice

As shown in Figure 2C, the body weight of the mice in the HC group increased steadily. The body weight of mice in the DSS group began to decrease on the 4th day after the establishment of the UC model, and the degree of weight loss increased on the 5th day (12.5%). On the 7th day, the weight loss of the DSS group was more than 28.9% compared with the HC group. In the PM group and the SPM group, the body weight fluctuated within the normal range in the first 4 days, and the weight decreased on the 5th day; this was relatively gentle compared with the DSS group. On the 7th day, the weight loss in the PM group and the SPM group was 23.9% and 15.0%, respectively, indicating that PPM60−III and SPPM60−III effectively prevented the trend of UC weight loss.

On the 4th day, the DSS, PM, and SPM groups showed a significant increase in the DAI score (Figure 2D), which was consistent with the significant weight loss of mice. The mice in each group had loose feces and even slightly bloody feces on the 4th day. The scores of the DAI in the PM and SPM groups were lower than that in the DSS group, and the score in the SPM group was much lower. At the end of the experiment, the average colon length of the HC group was 7.06 ± 0.82 cm, and the average colon length of the DSS group was 4.72 ± 1.22 cm. The average colon lengths of the PM group and the SPM group were 4.95 ± 1.15 cm and 5.62 ± 0.82 cm, respectively (Figure 2E,F). The length of the colon in the DSS group was significantly shorter than that in the HC group (*p <* 0.01). The condition of colonic shortening was improved subsequently in both the PM and SPM groups. SPPM60−III had a significant effect on the conditions of colonic shortening (*p <* 0.01).

**Figure 3:**
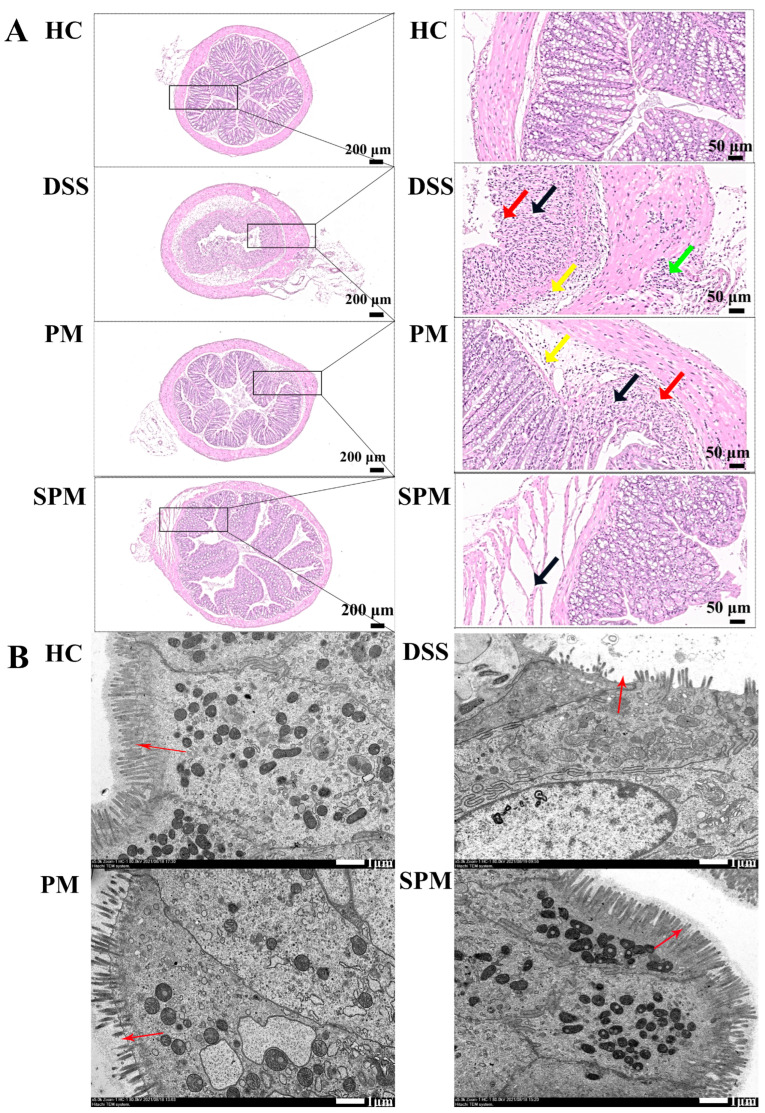
PPM60−III and SPPM60−III reduced colonic tissue. (**A**) Colon tissue section (HE staining): left, 4.5′; right, 20.0×; (**B**) Representative transmission electron microscopy images in colon tissues.

As shown in Figure 3A, in the HC group, the structure of each layer of colon tissue was clear. In the DSS group, diffuse ulcer, mucosal glandular necrosis, necrosis invading serosa, and large-area edema of submucosa were seen in the colonic mucosa (yellow arrow). In the PM group, there was necrosis of intestinal glands in ulcer mucosa and infiltration of inflammatory cells (black arrow), and edema of submucosa below the ulcer focus and infiltration of inflammatory cells (green arrow). In the SPM group, a small area of muscle layer was loosely arranged (black arrow), and no obvious inflammatory cell infiltration was found. As shown in Figure 3B, the microstructure of the HC group showed that the intestinal villi were normal, tightly arranged (red arrow), and the connections between the cells were intact; the microstructure of the DSS group showed that many of the intestinal villi were lost, with a large number of gaps (red arrow); the microstructure of the PM group showed that the loss of intestinal villi was significantly reduced, although some gaps (red arrows) can still be seen. The microstructure of the SPM group showed that the loss of intestinal villi was significantly reduced, and there were no obvious gaps (red arrows) visible.

### 3.3. Flow Cytometry Analysis and Determination of Inflammatory Cytokines

**Figure 4:**
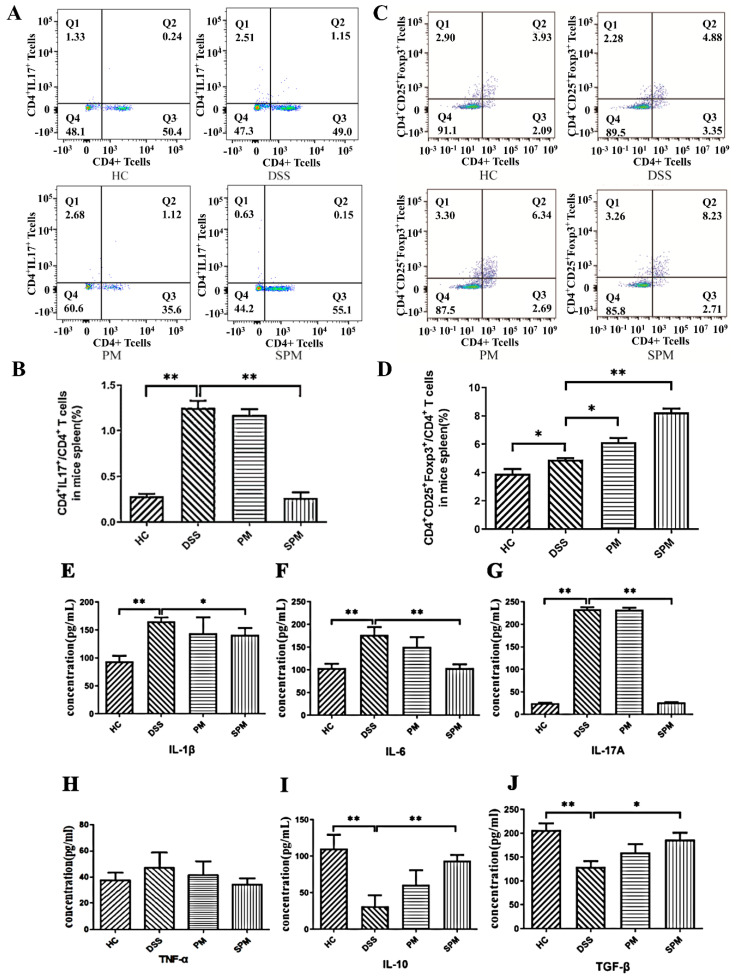
Changes in Th17 and Treg cells in spleen and cytokine content in colon of mice. (**A**) Th17 cell flow analysis(Q2); (**B**) percentage of Th17 cells (n = 10); (**C**) Treg cell flow analysis(Q2); (**D**) percentage of Treg cells; (**E**) IL−1β; (**F**) IL−6; (**G**) IL−17A; (**H**) TNF−a; (**I**) IL−10; (**J**) TGF−β. * *p* < 0.05, ** *p* < 0.01.

The results of the flow cytometry analysis showed that the percentages of CD4^+^IL-17A^+^T cells in CD4^+^T cells from mouse spleen in the HC, DSS, PM, and SPM groups were 0.28 ± 0.04%, 1.25 ± 0.11%, 1.17 ± 0.13%, and 0.26 ± 0.09%, respectively (Figure 4A,B). Compared with the DSS group, the SPM group decreased significantly (*p <* 0.01), close to the level of the HC group.

As shown in Figure 4C,D, the percentages of CD4^+^CD25^+^Foxp3^+^T cells in CD4^+^T cells in mouse spleen were 3.91 ± 0.59%, 4.92 ± 0.18%, 6.14 ± 0.54%, and 8.24 ± 0.51% in the HC, DSS, PM, and SPM groups, respectively. The percentages of Treg cells in the PM group (*p <* 0.05) and in the SPM group (*p* < 0.01) were significantly higher than that in the DSS group.

Compared with the HC group (Figure 4E–G), the contents of IL-1β, IL-6, and IL-17A in the colonic homogenate of UC mice increased significantly, indicating that severe inflammatory reaction occurred in colonic tissue of UC mice. The concentrations of IL-1β and IL-6 decreased in the PM group, and there was a significant difference in the SPM group (*p <* 0.01). TNF-α increased in the DSS group, and the levels of TNF-α in the PM and SPM groups had a big decrease (*p >* 0.05, Figure 4H). The trend in the amount of IL-17A is the same as the decreasing trend in the number of Th17 cells, indicating that PPM60−III and SPPM60−III inhibit the number of Th17 cells, and then, inhibit the production of IL-17A and the secretion of IL-1β and IL-6. Among them, the anti-inflammatory effect of SPPM60−III is better, indicating that sulfation could help improve the anti-inflammatory effect of pine pollen polysaccharide.

Compared with the HC group, the concentrations of IL-10 and TGF-β decreased in the DSS group (*p* < 0.01, Figure 4I,J). The concentrations of IL-10 and TGF-β increased in the PM group and SPM group, and increased significantly in the SPM group (*p* < 0.05, Figure 4I,J). IL-10 is secreted by Treg cells, and the trend is the same with Treg cells. Regulating the number of Treg cells contributes to the secretion of IL-10 and TGF-β, and has an anti-inflammatory effect.

### 3.4. Analysis of Serum ^1^H-NMR and Metabolic Pathway

**Figure 5:**
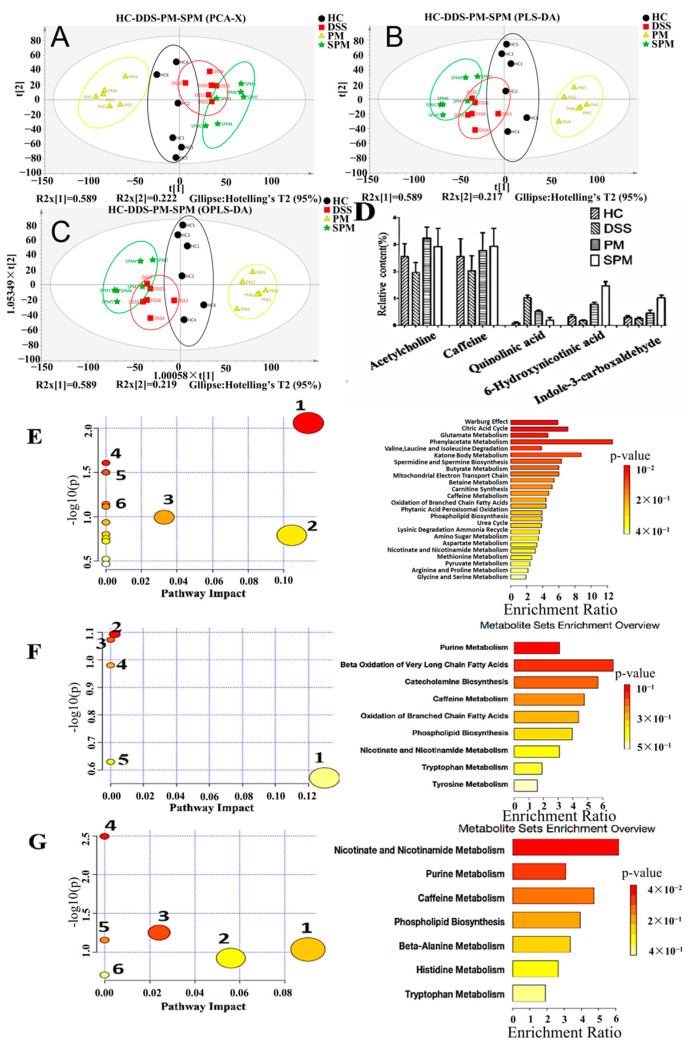
Analysis of serum nuclear magnetic resonance and differential metabolites in mice of HC group, DSS group, PM group, and SPM group. (**A**) PCA score chart; (**B**) PLS score chart; (**C**) OPLS score chart; (**D**) change trend chart of differential metabolites; (**E**) an overview of metabolic pathways and enrichment of differential metabolites in HC and DSS groups (1. alanine, aspartate, and glutamate metabolism; 2. aspartate and glutamate metabolism; 3. TCA; 4. aminoacyl-tRNA biosynthesis; 5. nitrogen metabolism; 6. arginine biosynthesis); (**F**) summary of metabolic pathway and enrichment of different metabolites in PM group and DSS group (1. tyrosine metabolism; 2. purine metabolism; 3. caffeine metabolism; 4. nicotinate and nicotinamide metabolism; 5. glycerophospholipid metabolism); (**G**) summary of metabolic pathway and enrichment of different metabolites in SPM group and DSS group (1. histidine metabolism; 2. alanine metabolism; 3. purine metabolism; 4. nicotinate and nicotinamide metabolism; 5. caffeine metabolism; 6. glycerophospholipid metabolism).

PCA analysis showed that there is obvious separation in each group (Figure 5A), and the differences within the groups are smaller than those between the groups, which can basically achieve separation. Figure 5B,C show the comparisons between the HC group, PM group, SPM group, and DSS group. PLS-DA and OPLS-DA analyses achieve complete separation between groups, and the replacement test is good.

According to the differential metabolite information between the HC group and DSS group in Table 2, compared with the HC group, there were nine differential metabolites with VIP values greater than 1.31. They were N, N-dimethylformamide, L-glutamine, succinic acid, L-methionine, undecanoic acid, alpha-lipoic acid, acetylcholine, cholesteryl sulfate, and caffeine. According to the information of differential metabolites between the PM group and DSS group in Table 3, nine differential metabolites of 6-hydroxynicotinic acid, glutaconic acid, dopamine, acetyl-L-carnitine, caffeine, xanthosine, acetylcholine, indole-3-carboxaldehyde, and quinolinic acid with VIP values greater than 1.15 were selected in the PM group. According to the differential metabolite information between the SPM group and DSS group in Table 4, compared with the DSS group, there were nine differential metabolites with VIPs greater than 1.59, which were 6-hydroxynicotinic acid, trigonelline, L-carnosine, nicotinic acid, caffeine, hypoxanthine, acetylcholine, indole-3-carboxaldehyde, and quinolinic acid in the SPM group. The common metabolites caffeine, acetylcholine, indole-3-formaldehyde, and 6-hydroxynicotinic acid increased significantly in the PM and SPM groups, while quinolinic acid decreased significantly.

As shown in Figure 5D, the common metabolites of the four groups were caffeine, acetylcholine, indole-3-formaldehyde, 6-hydroxynicotinic acid, and quinolinic acid, of which caffeine, acetylcholine, indole-3-formaldehyde, and 6-hydroxynicotinic acid were significantly higher than in the DSS group, while quinolinic acid decreased significantly compared with the DSS group.

As shown in Figure 5E, compared with the HC group, the ingestion of DSS changed the metabolic pathways of alanine, aspartate, and glutamate metabolism, cysteine and methionine metabolism, TCA (tricarboxylic acid cycle), aminoacyl-tRNA biosynthesis, nitrogen metabolism, and arginine biosynthesis. The enrichment overview map (Figure 5E) shows that it affected phenylacetic acid metabolism, alanine, aspartic acid, and glutamic acid metabolism, ketone body metabolism, TCA, spermine and spermine biosynthesis, and butyric acid metabolism. As shown in Figure 5F, compared with the HC group, the PM group changed the metabolic pathways of tyrosine metabolism, purine metabolism, caffeine metabolism, nicotinate and nicotinamide metabolism, and glycerophospholipid metabolism. The enrichment overview map shows that it affected purine metabolism, fatty acid β oxidation, caffeine metabolism, branched-chain fatty acid oxidation, phospholipid biosynthesis, nicotinic acid and nicotinamide metabolism, tryptophan metabolism, and tyrosine metabolism.

As shown in Figure 5G, compared with the HC group, the SPM group changed the metabolic pathways of histidine metabolism, alanine metabolism, purine metabolism, nicotinate and nicotinamide metabolism, caffeine metabolism, and glycerophospholipid metabolism. The enrichment overview map shows that it affected nicotinate and nicotinamide metabolism, purine metabolism, caffeine metabolism, histidine metabolism, β-alanine metabolism, and glycerophospholipid metabolism.

### 3.5. The Effects of PPM60−III and SPPM60−III on Intestinal Microflora of UC Mice

**Figure 6:**
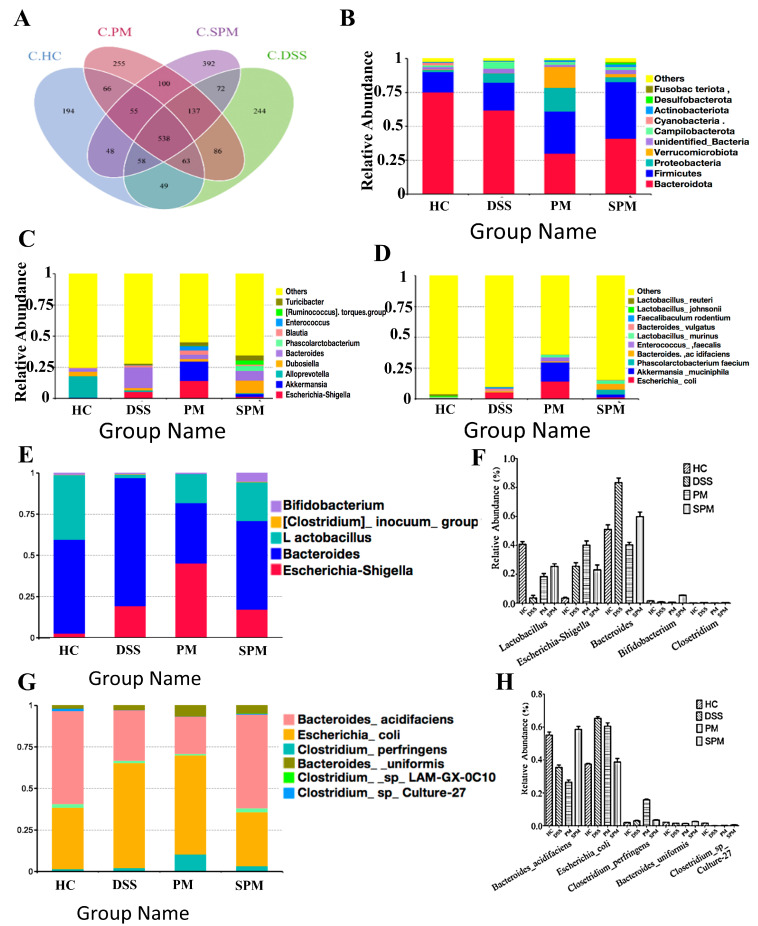
The abundance of intestinal flora in the mouse colon. (**A**) OTU Venn diagram of all groups; (**B**) relative abundance of species at the phylum taxonomic level; (**C**) relative abundance map of species at the genus taxonomic level; (**D**) relative abundance of species at the taxonomic level; (**E**) typical species at the genus taxonomic level; (**F**) relative abundance of typical species at the genus taxonomic level; (**G**) typical species at the taxonomic level; (**H**) relative abundance of typical species at the taxonomic level.

As shown in Figure 6A, the numbers of OTUs in the HC group, DSS group, PM group, and SPM group were 1071, 1247, 1300, and 1400, respectively. The number of OTUs shared by the four groups was 538. The numbers of OTUs specific to the HC group, DSS group, PM group, and SPM group were 194, 244, 255, and 392, respectively. The intestinal flora of mice in the HC group was stable. The number of OTUs in the DSS group increased. The increase in intestinal microflora diversity is a sign of the improvement of UC. Mice rely on their own immune system to resist UC. PPM60−III and SPPM60−III could restore normal intestinal homeostasis, and the diversity of intestinal flora increased significantly. As shown in Figure 6B in the HC group, *Firmicutes* (15.1%) and *Bacteroidetes* (75.1%) were the main bacteria, *Proteobacteria* was 1.5%, and *Verrucomicrobia* was 0.05%. The relative abundance of *Firmicutes* in the DSS group was 20.4%. Compared with HC group, the *Proteobacteria* increased significantly, by 5.4%. In the PM group, *Firmicutes* accounted for 31.0% and *Bacteroides* accounted for 30.0%. The relative abundance of *Proteobacteria* and *Verrucomicrobia* were roughly the same, at 17.5% and 15.5%, respectively. The proportions of the *Firmicutes* (41.8%) and *Bacteroides* (41.0%) in the SPM group were roughly the same; *Proteobacteria* accounted for 3.6%, and *Verrucomicrobia* accounted for 2.4%, which were roughly the same as the HC group, indicating that the intestinal flora in the colon were developing towards a normal level.

At the genus level(Figure 6C), the HC group is mainly composed of *Alloprevotella*, accounting for 17.4%, *Dubosiella* for 3.4%, *Akkermansia* for 0.48%, and *Bacteroides* for 2.9%, respectively. The bacterial genera with a high proportion in the DSS group included *Bacteroides* (16.6%), *Escherichia-Shigella* (5.2%), *Dubosiella* (2%), and *Blautia* (1.5%). The abundances of *Bacteroides* and *Broutella* were significantly increased in the DSS group. In the PM group, *Akkermansia* (15.5%) and *Escherichia coli* (14.2%) were significantly higher than in the DSS group. *Bacteroides* in the SPM group accounted for 7.8%; the abundance increased and gradually returned to near normal levels.

Figure 6D is the main bacterial species in the HC group are *Lactobacillus murine* and *Lactobacillus reuteri*. The main bacterial species in the DSS group are *Escherichia-Shigella*, *Bacteroides acidogenes*, and *Bacteroides vulgaris*. *Akkermansia*, *Enterococcus faecalis*, *Escherichia coli*, and *Lactobacillus* were significantly increased in the PM group, and the abundances of *Bacteroides acidogenes*, *Lactobacillus*, and *Enterococcus faecalis* in the SPM group were increased.

In the relative abundance diagram of typical species at the genus level (Figure 6E), in the HC group *Lactobacillus* accounted for 39% and *Bacteroides* accounted for 57%; *Escherichia coli* accounted for 2.7% and *Bifidobacterium* accounted for 1.0%. The main bacterial species in the DSS group were *Escherichia-Shigella,* accounting for 19.2%, and *Bacteroides*, accounting for 77.8%, with a significant increase. *Lactobacillus* accounted for 1.9% and *Bifidobacteria* 0.04%, with a significant decrease. In the PM group, *Bifidobacterium* accounted for 5.0%, with a significant increase, while *Bacteroides* accounted for 53.6%, gradually approaching normal levels.

It is obvious that PPM60−III and SPPM60−III had a protective effect on the intestinal flora of UC mice induced by DSS ingestion. In the relative abundance chart of typical species at the classification level (Figure 6F), *Bacteroides acidogenes*, *Escherichia coli*, *Clostridium perfringens*, *Escherichia coli*, and *Clostridium perfringens* accounted for 55.1%, 37.5%, 1.9%, 65.3%, and 3.1%, respectively, in the HC group. In the DSS group, *Escherichia coli* accounted for 65.3% and *Clostridium perfringens* accounted for 3.1%, increasing significantly, while *Bacteroides acidogenes* accounted for 35.5%, decreasing significantly.

In the PM group, *Escherichia coli* accounted for 60.4% and *Clostridium perfringens* accounted for 15.9%, significantly increasing, while *Bacteroides acidogenic* accounted for 35.5%, significantly decreasing. The percentages of *Bacteroides acidogenes*, *Escherichia coli*, and *Clostridium perfringens* in the SPM group were 58.6%, 38.8%, and 3.5%, respectively, which were similar to those in the HC group, indicating that the typical species returned to the normal level. In the colonic contents of mice, the beneficial flora were *Lactobacillus* and *Akkermansia bacteria*; and the overgrowth of *Escherichia coli* were shown to cause intestinal inflammation, the over reproduction of *Enterococcus faecalis* was also shown to lead to inflammatory infection (Figure 6G,H).

**Figure 7:**
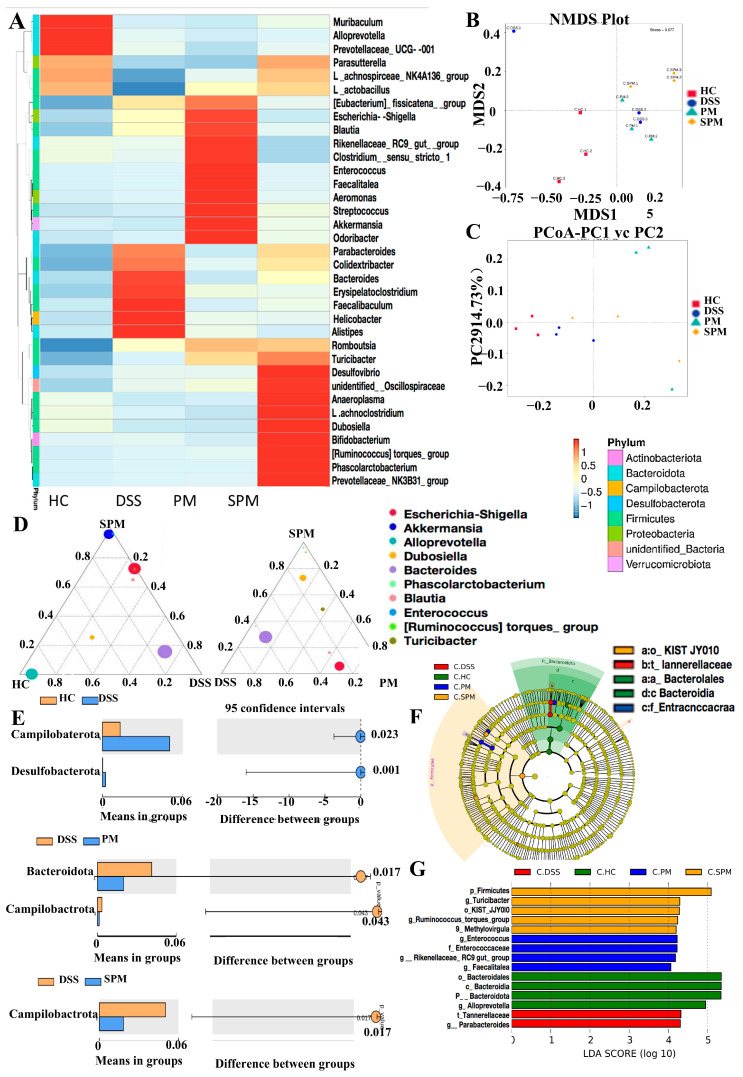
Correlation analysis diagram of intestinal flora in mouse colon. (**A**) Species abundance clustering heat map at the genus taxonomic level; (**B**) PCoA score map of mouse colonic intestinal flora based on weighted Unifrac distance; (**C**) PCoA score map of mouse colonic intestinal flora based on unweighted Unifrac distance; (**D**) ternary phase diagram of mouse colonic intestinal flora at the level of genus classification; (**E**) species difference between t-test groups of colonic intestinal flora; (**F**) LEfSe analysis cluster tree; (**G**) LDA value distribution histogram.

The intestinal flora heat map (Figure 7A) shows that the bacteria genera gathered in the HC group are *Muribaculum*, *Prevotellaceae*, *Prevotellaceae*, *Lachnospiraceae*, and *Parasutterella.* The abundance is concentrated in the phyla *Bacteroidetes* and *Firmicutes*; the genera gathered in the DSS group include *Parabacteroides*, *Helicobacter*, *Alisipes*, *Faecalitalea*, *Erysipelatoclostridium*, *Bacteroides*, and *Parabacteroides*; the abundance is concentrated in *Firmicutes* and Proteobacteria, *Campylobacteria* accounts for a large part. The bacteria in the PM group include *Akkermansia*, *Odoribacter*, *Streptococcus*, *Faebacterium*, *Aeromonas*, *Enterococcus*, *Rikenellaceae_RC9_gut_group*, *Clostridium*, *Escherichia*, *Eubacterium*, and *Blautia*; the abundance is concentrated in *Bacteroides*, Proteobacteria, and *Verrucomicrobia*. The bacterial genera gathered in the SPM group are *Phascolarctobacterium*, *Prevotella*, *Ruminococcus*, *Bifidobacterium*, *Dubosiella*, *Lachnoclostridium*, *Anaeroplasma*, *Desulfovibrio*, and *Turicibacter*; the abundance is concentrated in the phylua *Bacteroidetes*, *Firmicutes*, and *Actinomycetes*. As shown in Figure 7B,C, the HC group has a clearer difference. The PM group and the SPM group have a clear trend of separation from the DSS group, and the relative distance of separation is smaller than that of the HC group. The PM and SPM groups are farther away from the HC group, and the differences within the groups are greater, indicating that the individual differences between PPM60−III and SPPM60−III are more significant. Figure 7D is a ternary phase diagram at the genus taxonomic level. *Prevotella* tends to the HC group, and *Bacteroides* tends to the DSS group. *Escherichia coli* and *Akkermansia* tend to the PM group, and *Turicibacter* and *Ruminococcus* tend to the SPM group. Figure 7E shows that the intestinal flora has obviously different dominant species, *Campylobacterota* and *Desulfobacter*, which are obviously dominant in the DSS group, and the number is significantly increased. In the PM group, the number of *Bacteroides* decreases, and in the SPM group, the numbers of *Verrucomicrobia* and *Campylobacter* decrease.

Figure 7F is the LEfSe analysis cluster tree, and different species were found in Figure 7G. In the HC group, the representative different bacteria is *Bacteroidetes* at the phylum level, and *Prevotella* at the level of genus. In the DSS group, the representative difference at the family level is *Tannerellaceae*, and at the genus level is *Bacteroides*. The representative different bacteria in the SPM group are *Firmicutes* at the phylum level and *Kist-ijy010* at the order level, and *Turicibacter*, *Rumencoccus*, and *Methylovirgula* at the genus level. In the PM group, with representative different bacteria at the family level is *Enterococcus*, and at the genus level are *Rikenellaceae_RC9_gut_group*, *Enterococcus,* and *Neochiamydia*. These results indicate that PPM60−III and SPPM60−III could improve the disturbance of intestinal flora, and the protective effect of SPPM60−III on the intestinal flora composition has more advantages.

**Figure 8:**
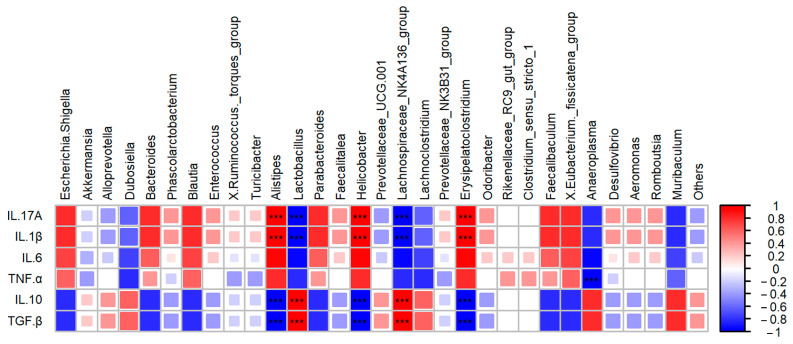
Heatmap for correlation analysis between gut microbiota and inflammatory factors. Red represents positive correlation, and blue represents negative correlation. *** *p* < 0.001.

Figure 8 is a heat map of the joint analysis of the correlation between the 30 bacterial taxa with the highest relative abundances at genus level and cytokine factors. It can be seen that the genera show a dual regulatory effect on anti-inflammatory factors and pro-inflammatory factors. *Alistipes*, *Helicobacter*, and *Erysipelatoclostridium*, with high abundance in the DSS group, significantly promoted the inflammatory process and showed a significant positive correlation with the pro-inflammatory cytokines IL-6 and IL-17A, and a significant negative correlation with the anti-inflammatory cytokines IL-10 and TGF-β (*p <* 0.001). On the contrary, *Lactobacillus* significantly recovered in the PM group, and *Lachnospiraceae_NK4A136_group*, the characteristic bacteria of the HC group, showed a significant negative correlation with the pro-inflammatory cytokines IL-6 and IL-17A, and a significant positive correlation with the anti-inflammatory cytokines IL-10 and TGF-β (*p* < 0.001). In addition, the characteristic bacteria *Anaeroplasma* in the SPM group showed a significant negative correlation with TNF-α.

## 4. Discussion

An acute UC animal model was constructed by using DSS drinking water. After the fourth day, the activity of the mice in the DSS group significantly reduced. Their food intake and water intake were also reduced, and the weights of the mice were reduced significantly. Loose stools and even bloody stools were identified. After the fifth day, the weight loss was less significant in the PM group and the SPM group, but the symptoms of stool and blood in the stool revealed in the SPM group were more significant (*p* < 0.05). In addition to weighing the mice every day, the DAI score was tested to evaluate the severity of the mice’s diseases [34]. The DAI score of the DSS group increased sharply on the fifth day, indicating that the mice had acute inflammation; the increase rates of the PM and SPM groups were lower than that of the DSS group, and the situation was significantly milder than that of the DSS group. The above results show that DSS-induced UC mice were successfully modeled; PPM60−III and SPPM60−III could prevent the UC from becoming more serious and restore inflammation to a certain extent, consistent with some other previous reports of polysaccharides. For example, *Lycium barbarum* polysaccharide is an effective component of *Lycium barbarum*, which can regulate Th17/Treg imbalance in UC mice induced by DSS, and improve the symptoms of UC mice [35]. *Aloe vera* polysaccharide is a biologically active substance extracted from plant aloe fluid, which prevents the release of pro-inflammatory cytokines such as IL−17A and IL−6 in colon tissue, and enhances anti-inflammatory cytokines such as IL−10, and TGF−β expression regulates the ratio of Th17/Treg cells and alleviates the symptoms of UC [36]. *Pinus massoniana* pollen polysaccharide alleviates colonic injury, relieves colitis symptoms in DSS−induced colitis mice, and increases the proportion and diversity of gut-beneficial bacteria in mice, which plays a decisive role in slowing down metabolic disorders and resisting inflammatory factors [37].

More and more reports have shown that Th17/Treg cells have a big relationship with UC [38,39,40]. The results of the Th17/Treg cells showed that compared with the HC group, the Th17/Treg cells of the DSS group were significantly imbalanced, and the secretion of pro-inflammatory cytokines increased. After intragastric administration of PPM60−III and SPPM60−III, the levels of IL-1β, IL-6, IL-17A, and TNF-α were decreased, the secretion of anti-inflammatory cytokines IL-10 and TGF-β increased, and the Th17/Treg balance was gradually restored. The improvement effect of SPPM60−III was significantly better than that of PPM60−III, as found by comparing the improvement effects of PPM60−III and SPPM60−III.

Metabolomics techniques have always been used to evaluate the efficacy of functional factors and study their mechanisms [38]. In this study, ^1^H−NMR experiments were conducted to screen the biomarkers of these groups, and then, explore the protective mechanism of PPM60−III and SPPM60−III on UC mice. The results of metabolomics showed that DSS induced the metabolic disorder of some amino acids (alanine, aspartate, glutamate, cysteine, methionine) and TCA (tricarboxylic acid cycle), and induced the synthesis disorder in aminoacyl-tRNA, nitrogen, and arginine biosynthesis. The disorder of amino acids in this experiment was consistent with previous studies [38]. Amino acids, that are the basic unit of protein molecular structure and function, are always found in the non-targeted metabolomics and inflammation markers of UC mice [38] and play an important role in the occurrence and development of UC inflammation [38]. To be specific, aspartic acid affects nerve regulation and improves liver function; glutamic acid can be converted into alanine through transamination; and alanine participates in the formation of pyruvate. TCA is inseparable from the participation of pyruvate. TCA provides an energy source. Pyruvate is an antagonist of peroxides, which can inhibit the damage of the intestinal mucosa caused by peroxides [39]. Pyruvate is also a key regulatory intersection of T cells’ glycolysis and oxidative metabolism [40]. Th17 cells rely on glutamate metabolism and glycolysis to obtain energy, convert alanine into lactic acid, generate large amounts of lactic acid, damage the intestinal mucosa, and trigger inflammation [41]. Treg cells promote the decomposition of pyruvate, enhance TCA, and rely on pyruvate metabolism for energy [42]. Inflammation is a process of consuming energy, and UC causes a large change in local energy metabolism. TCA is the regulation center of glucose, lipids, and amino acid metabolism. UC is inseparable from the supply of energy [43]. Arginine participates in normal physiological metabolism and is part of the ornithine cycle and affects the biosynthesis of aminoacyl-tRNA, and an increase in arginine causes the transformation of Treg cells from glycolysis to oxidative phosphorylation, promoting the production of Treg cells with stronger vitality and immune function [44]. When purine metabolism is abnormally increased, hypoxanthine is converted into xanthine under the action of oxidase, generating uric acid and causing inflammation [45]. UC can impair phospholipid metabolism [46]. Glycerophospholipid is a component of the cell membrane, participating in the recognition and secretion of inflammatory factors [47]. In the PM group and SPM group, the metabolic level of glycerophospholipids was restored, effectively inhibiting the development of UC. Niacin is converted to nicotinamide by transamination, which is the precursor of nicotinamide adenine dinucleotide [48]. In the PM group and SPM group, it increased the metabolic levels of nicotinate and nicotinamide, inhibited the NF-κB signaling pathway, and inhibited the release of pro-inflammatory cytokines [49]. Caffeine metabolism increases the release of neurotransmitters, has the functions of relieving pain and promoting digestion, and forms different dimethylxanthines. Paraxanthine is the main metabolite, as well as theobromine and chophylline [50]. Acetylcholine activates the nAChR/ERK pathway and promotes the secretion of IL-10. When IBD occurs, acetylcholine reveals the neuro-immune regulatory pathway, which may be a new target for treatment [51]. In the PM group and the SPM group, the content of acetylcholine increased, and the increase in the SPM group was more obvious, indicating that acetylcholine can have an anti-inflammatory effect during the development of inflammation and improve the condition of UC. Through analysis, it was found that in the process of UC, the metabolic pathways of alanine, aspartate, and glutamate metabolism, cysteine and methionine metabolism, TCA, aminoacyl-tRNA biosynthesis, nitrogen metabolism, and arginine biosynthesis changed in the DSS group. Acid biosynthesis underwent varying degrees of changes, resulting in an increase in Th17 cells, an increase in Treg cells, and an imbalance of Th17/Treg, leading to inflammation. In summary, PPM60−III regulates tyrosine metabolism, increases the proportion of Treg cells, and affects the Th17/Treg balance. SPPM60−III regulates histidine metabolism. Alanine metabolism significantly increases the proportion of Treg cells but significantly inhibits the proliferation of Th17 cells, making the Th17/Treg balance close to the level of the HC group. The restoration of purine metabolism and glycerophospholipid metabolism, and increases in nicotinate and nicotinamide metabolism and caffeine metabolism helped inhibit inflammation, causing an increase in the secretion of acetylcholine, improving the pathological condition of UC.

Disorders of intestinal flora play an important role in the pathogenesis of UC [52].The gastrointestinal tract provides a unique environment that maintains the diversity of microbial species and a tight ecosystem, that is, the intestinal flora [53]. *Firmicutes* and *Bacteroidetes* are important parts of healthy intestines, with relative abundance ranging from 66% to 98%, which play an important role in immune regulation [54]. The intestinal flora is stable and maintains a dynamic balance. When this balance is changed it causes changes in the species diversity of the intestinal flora. The decline in beneficial bacteria and the proliferation of harmful bacteria are important characteristics of UC [55], which further leads to damaged intestinal barriers and disrupted tight junctions between cells. After DSS intervention, the abundance of *Escherichia coli* increases, which is consistent with the culture results of fecal samples from patients with colitis [56]. *Escherichia coli* is a pro-inflammatory bacterium and shows adhesion and invasion behaviors in the intestine [57]. In vitro studies have shown that after the lumen of the small intestinal mucosa is exposed to intestinal bacteria such as *Escherichia coli*, the resistance of small intestinal tissue decreases, and the zonulin expression pathway is activated to induce the disintegration of tight junctions. At the same time, zonula occludens-1 protein (ZO-1) is detached from the tight junction complex, and the permeability of the tight junctions changes [58]. According to our study, pine pollen polysaccharides and sulfated polysaccharides can regulate the Th17/Treg balance, the expression levels of tight junction proteins and inflammatory factors, and inflammation-related pathways by upregulating the abundance of beneficial bacteria. As shown in the cluster heat map, the level of the representative genus Akkermansia in the PM group is significantly increased. Studies have shown that Akkermansia induces IgG1 production and antigen-specific T cell responses, playing a role in improving intestinal barriers. Similarly, *Lactobacillus* restores the balance of the intestinal immune system, induces the proliferation of Treg, improves the protective effect of the intestinal mucosa, and reduces the release of pro-inflammatory cytokines. The outbreak of UC causes a significant decrease in the types and number of lactobacilli. Ingestion of *Lactobacillus plantarum* L15 reduces UC and improves intestinal flora imbalance [59]. The increase in segmented filamentous bacteria significantly promotes the development and differentiation of T cells in the intestine [60,61]. The role of segmented filamentous bacteria has been determined and cytokine production tested by reconstituting sterile mice [62]. It tightly adheres to the intestinal mucosa, induces cell aggregation in the lamina propria [63], and stimulates the intestinal immune response process, including the secretion of cytokines and chemokines, antimicrobial peptides, and serum amyloid and induces Th17 cell differentiation [64,65].

Certain bacteria promote Treg differentiation or stimulate IL-10 expression to enhance the anti-inflammatory effect of the immune system [64,66]. Intestinal commensal *Alistipes* is a key bacterium with pro-inflammatory properties in the carcinogenesis of colorectal cancer (CRC). In the fecal samples of patients with intestinal cancer, the significantly increased relative abundance of *Alistipes* is positively correlated with inflammatory factor signal transduction. TNF-α and IL-6 related to *Alistipes* may activate LRG1/TGF-β1 signal transduction, leading to the carcinogenesis of CRC [67]. The mixed colonization of *Clostridium IV* and *Cluster IV XIVa* strains of *Clostridium* originally isolated from mouse feces activated the phenomenon of Treg proliferation and differentiation [68]. The role of *Helicobacter* in ulcerative colitis (UC) has always been controversial [69]. Our results show that it is significantly positively correlated with IL-6 and IL-17A and weakens the expression of IL-10 and TGF-β. Enterotoxigenic *Bacteroides fragilis* is a special strain of *Bacteroides fragilis* [70]. It induces the production of IL-17 in the colon, increases the secretion of inflammatory factors, and triggers intestinal cancer [71]. The production and development of intestinal flora in Th17 cells provide immune signals and stimulate natural immune cells to secrete IL-6, IL-23, and IL-1β [72]. Decreases in *Lactobacillus* and *Bifidobacteria* [73] and significant increases in *Escherichia coli* and Bacteroides are clinical signs of a UC inflammation outbreak [74]. *Escherichia coli* aggravates the inflammatory response of UC induced by DSS [75]. The use of a mixture of five probiotics (*L. acidophilus*, *L. casei*, *Lactobacillus reuteri*, *Bifidobacterium bifidium*, and *Streptococcus thermophilus*) can induce the production of Treg cells in mice, increase the cell activity, and improve the immune response [76]. Using gavage probiotic *Bifidobacterium bifidum ATCC 29521* to treat DSS-induced UC mice up-regulates active oxygen-scavenging enzymes and inhibits the expression of inflammatory cytokines, down-regulates pro-inflammatory cytokines, inhibits the inflammatory pathway of NF-κB, and restores the intestine’s Dysflora dysbiosis, exerting its probiotic effect [77]. PM promotes the expression of IL-10 and TGF-β by increasing the abundance of *Lactobacillus*, providing further evidence for UC treatment methods targeting the intestinal flora, and previous research has shown that decreased *Ruminococcus* and *Lachnospiraceae* and increased *Enterobacteriaceae* correlate with a Treg/Th17 imbalance [78]. As the characteristic bacteria of the HC group, *Lachnospiraceae_NK4A136_group* shows a significant correlation with inflammatory cytokines and the characteristic bacteria *Anaeroplasma* of the SPM group is not only significantly correlated with the expression of cytokines, but can also delay the inflammatory process of allergic asthma by regulating tryptophan metabolism [79].

In addition, changes in the metabolite level of the flora are also important factors leading to the aggravation of the course of colitis. In addition to the roles mentioned above, *Akkermansia* can also regulate the level of short-chain fatty acids (SCFA) by participating in the production of propionate [80]. Propionate has potential health-promoting effects, including anti-lipogenesis, cholesterol reduction, and anti-inflammatory and anti-cancer effects [81]. As a representative genus of the SPM group, *Ruminococcus* can also promote the production of propionate [80] and regulate secondary bile acid metabolism [82], which may be related to the extraintestinal manifestations of ulcerative colitis [83].

With increases in bacteria such as *Escherichia coli*, *Escherichia-Shigella* in the DSS group, the original balance was broken. In the PM group, the abundance of *Akkermansia* and *Escherichia coli* were up-regulated, and the number of *Clostridium perfringens* increased significantly. In the SPM group, the abundance of *Bacteroides acidogenes* and *Escherichia coli* were restored. The abundance of *Fidobacteria* was up-regulated. SPM and PM both had the effect of improving the imbalance in the intestinal flora of mice, regulating and reducing the abundance of harmful flora, increasing the types and quantity of beneficial flora, and restoring and increasing the proportion of beneficial flora.

## 5. Conclusions

All in all, PPM60−III and SPPM60−III regulate the Th17/Treg balance, thereby inhibiting the secretion of pro-inflammatory cytokines, stimulating and increasing the secretion of related anti-inflammatory cytokines, and regulating the dynamic balance of intestinal flora. They led to a significant improvement in DSS-induced UC mice, and SPPM60−III had a better improvement effect.

## Figures and Tables

**Table 1:** DAI scoring criteria.

DAI Score	Weight Loss	Stool Consistency	Occult/Gross Bleeding
0	0	Normal	Normal
1	1~5%		
2	5~10%	Loose	Guiac (+)
3	10~15%		
4	>15%	Diarrhoea	Gross bleeding

Disease Activity Index = (combined score of weight loss, stool consistency and bleeding)/3. Normal stools = well-formed pellets; loose = pasty stools which do not stick to the anus; diarrhoea = liquid stools that stick to the anus.

**Table 2:** Differential metabolite information between HC group and DSS group.

Name	Shifts	VIP	Trend
N, N-Dimethylformamide	7.920 (s), 3.010 (d)	1.34	↑
L-Glutamine	3.766 (t), 2.446 (m), 2.125 (m)	1.34	↑
Succinic acid	2.393 (s)	1.32	↑
L-Methionine	3.851 (dd), 2.631 (t), 2.157 (m)	1.31	↑
Undecanoic acid	1.527 (t), 1.270 (t), 0.850 (t)	1.32	↑
Alpha-lipoic acid	3.585 (m), 3.197 (m), 3.128 (m), 2.478 (m), 2.387 (t)	1.31	↑
Acetylcholine	3.75 (t), 3.23 (s)	1.31	↑
Cholesteryl sulfate	5.280 (m), 3.840 (m), 2.373 (m), 0.651 (m)	1.32	↑
Caffeine	7.510 (s), 3.410 (s), 4.000 (s)	1.31	↓

“↑” represents that this substance is upregulated in the DSS group compared with the HC group, “↓” represents downregulation. “s” represents a singlet peak, “d” represents a doublet peak, “dd” represents two doublet peaks, “t” represents a triplet peak, “dt” represents two triplet peaks, and “m” represents a multiplet peak.

**Table 3:** Differential metabolite information between PM group and DSS group.

Name	Shifts	VIP	Trend
6-Hydroxynicotinic acid	8.083 (d), 6.604 (d)	1.16	↑
Glutaconic acid	6.651 (dt), 5.858 (dt), 3.073 (dd)	1.16	↑
Dopamine	6.900 (d), 6.748 (d), 2.867 (t)	1.16	↑
Acetyl-L-carnitine	2.13 (dd), 2.61 (dd), 3.61 (d)	1.16	↑
Caffeine	7.510 (s), 3.410 (s), 4.000 (s)	1.16	↑
Xanthosine	7.871 (s), 5.845 (d), 4.690 (t), 4.385 (dd), 3.817 (m)	1.15	↑
Acetylcholine	3.75 (t), 3.23 (s)	1.16	↑
Indole-3-carboxaldehyde carboxaldehyde	5.280 (m), 3.840 (m), 2.373 (m), 0.651 (m)	1.16	↑
Quinolinic acid	7.43 (dd), 8.00 (dd), 8.44 (dd)	1.16	↓

“↑” represents that this substance is upregulated in the PM group compared with the DSS group, “↓” represents downregulation. “s” represents a singlet peak, “d” represents a doublet peak, “dd” represents two doublet peaks, “t” represents a triplet peak, “dt” represents two triplet peaks, and “m” represents a multiplet peak.

**Table 4:** Differential metabolite information between SPM group and DSS group.

Name	Shifts	VIP	Trend
6-Hydroxynicotinic acid	8.083 (d), 6.604 (d)	1.62	↑
Trigonelline	9.114 (s), 8.826 (m), 8.072 (m), 4.428 (s)	1.61	↑
L-Carnosine	8.015 (s), 4.466 (q), 3.216 (t), 3.095 (m), 2.672 (q)	1.60	↑
Nicotinic acid	8.927 (s), 8.593 (d) 8.235 (t), 7.504 (d)	1.60	↑
Caffeine	7.510 (s), 3.410 (s), 4.000 (s)	1.60	↑
Hypoxanthine	8.200 (s), 8.170 (s)	1.61	↑
Acetylcholine	3.75 (t), 3.23 (s)	1.59	↑
Indole-3-carboxaldehyde	5.280 (m), 3.840 (m), 2.373 (m), 0.651 (m)	1.62	↑
Quinolinic acid	7.43 (dd), 8.00 (dd), 8.44 (dd)	1.61	↓

**“↑” represents that this substance is upregulated in the** SPM **group compared with the** DSS **group, “↓” represents downregulation.** “s” represents a singlet peak, “d” represents a doublet peak, “dd” represents two doublet peaks, “t” represents a triplet peak, “dt” represents two triplet peaks, and “m” represents a multiplet peak. “s” represents a singlet peak, “d” represents a doublet peak, “dd” represents two doublet peaks, “t” represents a triplet peak, “dt” represents two triplet peaks, and “m” represents a multiplet peak.

## Data Availability

The original contributions presented in the study are included in the article, further inquiries can be directed to the corresponding author.

## References

[1] Ungaro R., Mehandru S., Allen P.B., Peyrin-Biroulet L., Colombel J.F. (2017). Ulcerative colitis. Lancet.

[2] Fumery M., Singh S., Dulai P.S., Gower-Rousseau C., Peyrin-Biroulet L., Sandborn W.J. (2018). Natural History of Adult Ulcerative Colitis in Population-based Cohorts: A Systematic Review. Clin. Gastroenterol. Hepatol..

[3] Rubin D.T., Ananthakrishnan A.N., Siegel C.A., Sauer B.G., Long M.D. (2019). ACG Clinical Guideline: Ulcerative Colitis in Adults. Am. J. Gastroenterol..

[4] Abdalla M., Landerholm K., Andersson P., Andersson R.E., Myrelid P. (2017). Risk of Rectal Cancer After Colectomy for Patients With Ulcerative Colitis: A National Cohort Study. Clin. Gastroenterol. Hepatol..

[5] Nishida A., Inoue R., Inatomi O., Bamba S., Naito Y., Andoh A. (2018). Gut microbiota in the pathogenesis of inflammatory bowel disease. Clin. J. Gastroenterol..

[6] Luo X., Yue B., Yu Z., Ren Y., Zhang J., Ren J., Wang Z., Dou W. (2020). Obacunone Protects Against Ulcerative Colitis in Mice by Modulating Gut Microbiota, Attenuating TLR4/NF-κB Signaling Cascades, and Improving Disrupted Epithelial Barriers. Front. Microbiol..

[7] Bianchi E., Rogge L. (2019). The IL-23/IL-17 pathway in human chronic inflammatory diseases-new insight from genetics and targeted therapies. Genes Immun..

[8] de Jong R.J., Ohnmacht C. (2019). Defining Dysbiosis in Inflammatory Bowel Disease. Immunity.

[9] Gui X., Li J., Ueno A., Iacucci M., Qian J., Ghosh S. (2018). Histopathological Features of Inflammatory Bowel Disease are Associated With Different CD4^+^ T Cell Subsets in Colonic Mucosal Lamina Propria. J. Crohn’s Colitis.

[10] Luo A., Leach S.T., Barres R., Hesson L.B., Grimm M.C., Simar D. (2017). The Microbiota and Epigenetic Regulation of T Helper 17/Regulatory T Cells: In Search of a Balanced Immune System. Front. Immunol..

[11] Cui H., Cai Y., Wang L., Jia B., Li J., Zhao S., Chu X., Lin J., Zhang X., Bian Y. (2018). Berberine Regulates Treg/Th17 Balance to Treat Ulcerative Colitis Through Modulating the Gut Microbiota in the Colon. Front. Pharmacol..

[12] Liu C., Li Y., Chen Y., Huang S., Wang X., Luo S., Su Y., Zhou L., Luo X. (2020). Baicalein Restores the Balance of Th17/Treg Cells via Aryl Hydrocarbon Receptor to Attenuate Colitis. Mediat. Inflamm..

[13] Moschen A.R., Tilg H., Raine T. (2019). IL-12, IL-23 and IL-17 in IBD: Immunobiology and therapeutic targeting. Nat. Rev. Gastroenterol. Hepatol..

[14] Chizzolini C., Dufour A.M., Brembilla N.C. (2018). Is there a role for IL-17 in the pathogenesis of systemic sclerosis?. Immunol. Lett..

[15] Mohammadnia-Afrouzi M., Hosseini A.Z., Khalili A., Abediankenari S., Amari A., Aghili B., Nataj H.H. (2016). Altered microRNA Expression and Immunosuppressive Cytokine Production by Regulatory T Cells of Ulcerative Colitis Patients. Immunol. Investig..

[16] Zhu L., Shi T., Zhong C., Wang Y., Chang M., Liu X. (2017). IL-10 and IL-10 Receptor Mutations in Very Early Onset Inflammatory Bowel Disease. Gastroenterol. Res..

[17] Fantini M.C., Monteleone G. (2017). Update on the Therapeutic Efficacy of Tregs in IBD: Thumbs up or Thumbs down?. Inflamm. Bowel Dis..

[18] Wang X., Li D., Zhang Y., Wu S., Tang F. (2018). Costus root granules improve ulcerative colitis through regulation of TGF-β mediation of the PI3K/AKT signaling pathway. Exp. Ther. Med..

[19] Ihara S., Hirata Y., Koike K. (2017). TGF-β in inflammatory bowel disease: A key regulator of immune cells, epithelium, and the intestinal microbiota. J. Gastroenterol..

[20] Clough J.N., Omer O.S., Tasker S., Lord G.M., Irving P.M. (2020). Regulatory T-cell therapy in Crohn’s disease: Challenges and advances. Gut.

[21] Ding Y., Ding K., Tan Y., Huang S., Li M., Cai M., Song Y., Zhang S. (2019). Effect of Kuijie Gailiang Prescription Regulating Th17/Treg Balance on Intestinal Inflammatory Response in DSS Mice. J. Nanjing Univ. Tradit. Chin. Med..

[22] Song Y. (2020). Based on the Intestinal Flora and Treg/TH17 to Explore the Mechanism of Kuijie Enema Fluid II to Improve the Intestinal Inflammatory Response in DSS Mice. Master’s Thesis.

[23] Li P., Wei R., Wu Z., Zhang D. (2017). Function and Research Progress of Th17 /Treg Cells and Their Related Cytokines in Pathogenesis of Ulcerative Colitis. World Sci. Technol. Res. Dev..

[24] Yao J., Wei C., Wang J.Y., Zhang R., Li Y.X., Wang L.S. (2015). Effect of resveratrol on Treg/Th17 signaling and ulcerative colitis treatment in mice. World J. Gastroenterol..

[25] Gong Y., Lin Y., Zhao N., He X., Lu A., Wei W., Jiang M. (2016). The Th17/Treg Immune Imbalance in Ulcerative Colitis Disease in a Chinese Han Population. Mediat. Inflamm..

[26] Chang Z., Liu Q., Li Q. (2020). Research progress on function of pine pollen and its processing and utilization. Cereals Oils.

[27] Geng Y., Xing L., Sun M., Su F. (2016). Immunomodulatory effects of sulfated polysaccharides of pine pollen on mouse macrophages. Int. J. Biol. Macromol..

[28] Chu H.L., Mao H., Feng W., Liu J.W., Geng Y. (2013). Effects of sulfated polysaccharide from Masson pine (*Pinus massoniana*) pollen on the proliferation and cell cycle of HepG2 cells. Int. J. Biol. Macromol..

[29] Wang X., Sun Y., Zhao Y., Ding Y., Zhang X., Kong L., Li Z., Guo Q., Zhao L. (2016). Oroxyloside prevents dextran sulfate sodium-induced experimental colitis in mice by inhibiting NF-κB pathway through PPARγactivation. Biochem. Pharmacol..

[30] Murano M., Maemura K., Hirata I., Toshina K., Nishikawa T., Hamamoto N., Sasaki S., Saitoh O., Katsu K. (2000). Therapeutic effect of intracolonically administered nuclear factor kappa B (p65) antisense oligonucleotide on mouse dextran sulphate sodium (DSS)-induced colitis. Clin. Exp. Immunol..

[31] Sawa S., Lochner M., Satoh-Takayama N., Dulauroy S., Bérard M., Kleinschek M., Cua D., Di Santo J.P., Eberl G. (2011). RORγt+ innate lymphoid cells regulate intestinal homeostasis by integrating negative signals from the symbiotic microbiota. Nat. Immunol..

[32] Ma S., Chang H., Cheng D., Li H., Li L., Li X., Lu Y. (2018). Serum metabolomics reasearch of Tiansi Liquid on chronic stress rats utilizing NMR metabolomics. Chin. Tradit. Herb. Drugs.

[33] Wu S., Zhang L., Gong M., Wang S., Liang S., Zou Z. (2018). Metabonomic Study on Acute Hepatic Injury Induced by Diosbulbin B in Mice. China Pharm..

[34] Zhou H., Zhang H., Wang Y., Sun M. (2017). Efficacy and related mechanism of Saccharomyces boulardii on experimental colitis in mice. Chin. J. Dig..

[35] Huang W. (2017). Effect of LBP on the Balance of Th1/Th2 and Th17/Treg Cells in DSS Induced Colitis Mice. Master’s Thesis.

[36] Fu Z., Wang L., Ge H. (2019). Experimental study of the aloe polysaccharides relieving enteritis by regulating Th17/Treg cell balance. Chin. Arch. Gen. Surg. (Electron. Ed.).

[37] Niu X., Shang H., Chen S., Chen R., Huang J., Miao Y., Cui W., Wang H., Sha Z., Peng D. (2021). Effects of *Pinus massoniana* pollen polysaccharides on intestinal microenvironment and colitis in mice. Food Funct..

[38] Daniluk U., Daniluk J., Kucharski R., Kowalczyk T., Pietrowska K., Samczuk P., Filimoniuk A., Kretowski A., Lebensztejn D., Ciborowski M. (2019). Untargeted Metabolomics and Inflammatory Markers Profiling in Children With Crohn’s Disease and Ulcerative Colitis-A Preliminary Study. Inflamm. Bowel Dis..

[39] Kolho K.L., Pessia A., Jaakkola T., de Vos W.M., Velagapudi V. (2017). Faecal and Serum Metabolomics in Paediatric Inflammatory Bowel Disease. J. Crohns Colitis.

[40] Joseph A.M., Monticelli L.A., Sonnenberg G.F. (2018). Metabolic regulation of innate and adaptive lymphocyte effector responses. Immunol. Rev..

[41] Cluxton D., Petrasca A., Moran B., Fletcher J.M. (2019). Differential Regulation of Human Treg and Th17 Cells by Fatty Acid Synthesis and Glycolysis. Front. Immunol..

[42] Gerriets V.A., Kishton R.J., Nichols A.G., Macintyre A.N., Inoue M., Ilkayeva O., Winter P.S., Liu X., Priyadharshini B., Slawinska M.E. (2015). Metabolic programming and PDHK1 control CD4+ T cell subsets and inflammation. J. Clin. Investig..

[43] Fitzpatrick M., Young S.P. (2013). Metabolomics—A novel window into inflammatory disease. Swiss Med. Wkly..

[44] Geiger R., Rieckmann J.C., Wolf T., Basso C., Feng Y., Fuhrer T., Kogadeeva M., Picotti P., Meissner F., Mann M. (2016). L-Arginine Modulates T Cell Metabolism and Enhances Survival and Anti-tumor Activity. Cell.

[45] Baldissera M.D., Souza C.F., Doleski P.H., Zeppenfeld C.C., Descovi S., Da Silva A.S., Baldisserotto B. (2018). Xanthine oxidase activity exerts pro-oxidative and pro-inflammatory effects in serum of silver catfish fed with a diet contaminated with aflatoxin B_1_. J. Fish Dis..

[46] Martin F.P., Rezzi S., Philippe D., Tornier L., Messlik A., Hölzlwimmer G., Baur P., Quintanilla-Fend L., Loh G., Blaut M. (2009). Metabolic assessment of gradual development of moderate experimental colitis in IL-10 deficient mice. J. Proteome Res..

[47] Stock C., Schilling T., Schwab A., Eder C. (2006). Lysophosphatidylcholine stimulates IL-1 beta release from microglia via a P2X7 receptor-independent mechanism. J. Immunol..

[48] Penberthy W.T. (2009). Nicotinamide adenine dinucleotide biology and disease. Curr. Pharm. Des..

[49] Ma Y., Bao Y., Wang S., Li T., Chang X., Yang G., Meng X. (2016). Anti-Inflammation Effects and Potential Mechanism of Saikosaponins by Regulating Nicotinate and Nicotinamide Metabolism and Arachidonic Acid Metabolism. Inflammation.

[50] Caubet M.S., Elbast W., Dubuc M.C., Brazier J.L. (2002). Analysis of urinary caffeine metabolites by HPLC-DAD: The use of metabolic ratios to assess CYP1A2 enzyme activity. J. Pharm. Biomed. Anal..

[51] Zheng W., Song H., Luo Z., Wu H., Chen L., Wang Y., Cui H., Zhang Y., Wang B., Li W. (2021). Acetylcholine ameliorates colitis by promoting IL-10 secretion of monocytic myeloid-derived suppressor cells through the nAChR/ERK pathway. Proc. Natl. Acad. Sci. USA.

[52] Chen Y., Zhang L., Hong G., Huang C., Qian W., Bai T., Song J., Song Y., Hou X. (2020). Probiotic mixtures with aerobic constituent promoted the recovery of multi-barriers in DSS-induced chronic colitis. Life Sci..

[53] Karl J.P., Hatch A.M., Arcidiacono S.M., Pearce S.C., Pantoja-Feliciano I.G., Doherty L.A., Soares J.W. (2018). Effects of Psychological, Environmental and Physical Stressors on the Gut Microbiota. Front. Microbiol..

[54] Chen D., Wu J., Jin D., Wang B., Cao H. (2019). Fecal microbiota transplantation in cancer management: Current status and perspectives. Int. J. Cancer.

[55] Pei L.Y., Ke Y.S., Zhao H.H., Liu W.Z., Wang L., Jia C., Shi M.N., Fu Q.H., Cui J., Li S.C. (2019). Regulatory effect of Garidisan on dysbiosis of the gut microbiota in the mouse model of ulcerative colitis induced by dextran sulfate sodium. BMC Complement. Altern. Med..

[56] Dahal R.H., Kim S., Kim Y.K., Kim E.S., Kim J. (2023). Insight into gut dysbiosis of patients with inflammatory bowel disease and ischemic colitis. Front. Microbiol..

[57] Ciccia F., Guggino G., Rizzo A., Alessandro R., Luchetti M.M., Milling S., Saieva L., Cypers H., Stampone T., Di Benedetto P. (2017). Dysbiosis and zonulin upregulation alter gut epithelial and vascular barriers in patients with ankylosing spondylitis. Ann. Rheum. Dis..

[58] El Asmar R., Panigrahi P., Bamford P., Berti I., Not T., Coppa G.V., Catassi C., Fasano A. (2002). Host-dependent zonulin secretion causes the impairment of the small intestine barrier function after bacterial exposure. Gastroenterology.

[59] Wu M., Li P., An Y., Ren J., Yan D., Cui J., Li D., Li M., Wang M., Zhong G. (2019). Phloretin ameliorates dextran sulfate sodium-induced ulcerative colitis in mice by regulating the gut microbiota. Pharmacol. Res..

[60] Li F., Han Y., Cai X., Gu M., Sun J., Qi C., Goulette T., Song M., Li Z., Xiao H. (2020). Dietary resveratrol attenuated colitis and modulated gut microbiota in dextran sulfate sodium-treated mice. Food Funct..

[61] Wu H.J., Ivanov I.I., Darce J., Hattori K., Shima T., Umesaki Y., Littman D.R., Benoist C., Mathis D. (2010). Gut-residing segmented filamentous bacteria drive autoimmune arthritis via T helper 17 cells. Immunity.

[62] Gaboriau-Routhiau V., Rakotobe S., Lécuyer E., Mulder I., Lan A., Bridonneau C., Rochet V., Pisi A., De Paepe M., Brandi G. (2009). The key role of segmented filamentous bacteria in the coordinated maturation of gut helper T cell responses. Immunity.

[63] Round J.L., Mazmanian S.K. (2010). Inducible Foxp3^+^ regulatory T-cell development by a commensal bacterium of the intestinal microbiota. Proc. Natl. Acad. Sci. USA.

[64] Ivanov I.I., Atarashi K., Manel N., Brodie E.L., Shima T., Karaoz U., Wei D., Goldfarb K.C., Santee C.A., Lynch S.V. (2009). Induction of intestinal Th17 cells by segmented filamentous bacteria. Cell.

[65] Atarashi K., Nishimura J., Shima T., Umesaki Y., Yamamoto M., Onoue M., Yagita H., Ishii N., Evans R., Honda K. (2008). ATP drives lamina propria T(H)17 cell differentiation. Nature.

[66] Round J.L., Lee S.M., Li J., Tran G., Jabri B., Chatila T.A., Mazmanian S.K. (2011). The Toll-like receptor 2 pathway establishes colonization by a commensal of the human microbiota. Science.

[67] Fu J., Li G., Li X., Song S., Cheng L., Rui B., Jiang L. (2024). Gut commensal Alistipes as a potential pathogenic factor in colorectal cancer. Discov. Oncol..

[68] Abraham C., Cho J.H. (2009). IL-23 and autoimmunity: New insights into the pathogenesis of inflammatory bowel disease. Annu. Rev. Med..

[69] Liu W., Jiang Q., Xue S., Hui W., Kong W., Zhang M., Gao F. (2024). Clinical characteristics of ulcerative colitis patients with different types of Helicobacter pylori infection. Microbiol. Spectr..

[70] Chung L., Orberg E.T., Geis A.L., Chan J.L., Fu K., DeStefano Shields C.E., Dejea C.M., Fathi P., Chen J., Finard B.B. (2018). *Bacteroides fragilis* Toxin Coordinates a Pro-carcinogenic Inflammatory Cascade via Targeting of Colonic Epithelial Cells. Cell Host Microbe.

[71] Dejea C.M., Fathi P., Craig J.M., Boleij A., Taddese R., Geis A.L., Wu X., DeStefano Shields C.E., Hechenbleikner E.M., Huso D.L. (2018). Patients with familial adenomatous polyposis harbor colonic biofilms containing tumorigenic bacteria. Science.

[72] Omenetti S., Pizarro T.T. (2015). The Treg/Th17 Axis: A Dynamic Balance Regulated by the Gut Microbiome. Front. Immunol..

[73] Keshteli A.H., Millan B., Madsen K.L. (2017). Pretreatment with antibiotics may enhance the efficacy of fecal microbiota transplantation in ulcerative colitis: A meta-analysis. Mucosal Immunol..

[74] Chen G.L., Zhang Y., Wang W.Y., Ji X.L., Meng F., Xu P.S., Yang N.M., Ye F.Q., Bo X.C. (2017). Partners of patients with ulcerative colitis exhibit a biologically relevant dysbiosis in fecal microbial metacommunities. World J. Gastroenterol..

[75] Yang H., Mirsepasi-Lauridsen H.C., Struve C., Allaire J.M., Sivignon A., Vogl W., Bosman E.S., Ma C., Fotovati A., Reid G.S. (2020). Ulcerative Colitis-associated *E. coli* pathobionts potentiate colitis in susceptible hosts. Gut Microbes.

[76] Kwon H.K., Lee C.G., So J.S., Chae C.S., Hwang J.S., Sahoo A., Nam J.H., Rhee J.H., Hwang K.C., Im S.H. (2010). Generation of regulatory dendritic cells and CD4^+^Foxp3^+^ T cells by probiotics administration suppresses immune disorders. Proc. Natl. Acad. Sci. USA.

[77] Din A.U., Hassan A., Zhu Y., Zhang K., Wang Y., Li T., Wang Y., Wang G. (2020). Inhibitory effect of *Bifidobacterium bifidum* ATCC 29521 on colitis and its mechanism. J. Nutr. Biochem..

[78] Han L., Jin H., Zhou L., Zhang X., Fan Z., Dai M., Lin Q., Huang F., Xuan L., Zhang H. (2018). Intestinal Microbiota at Engraftment Influence Acute Graft-Versus-Host Disease via the Treg/Th17 Balance in Allo-HSCT Recipients. Front. Immunol..

[79] Wang H., He Y., Dang D., Zhao Y., Zhao J., Lu W. (2024). Gut Microbiota-Derived Tryptophan Metabolites Alleviate Allergic Asthma Inflammation in Ovalbumin-Induced Mice. Foods.

[80] Reichardt N., Duncan S.H., Young P., Belenguer A., McWilliam Leitch C., Scott K.P., Flint H.J., Louis P. (2014). Phylogenetic distribution of three pathways for propionate production within the human gut microbiota. ISME J..

[81] Hosseini E., Grootaert C., Verstraete W., Van de Wiele T. (2011). Propionate as a health-promoting microbial metabolite in the human gut. Nutr. Rev..

[82] Kakiyama G., Pandak W.M., Gillevet P.M., Hylemon P.B., Heuman D.M., Daita K., Takei H., Muto A., Nittono H., Ridlon J.M. (2013). Modulation of the fecal bile acid profile by gut microbiota in cirrhosis. J. Hepatol..

[83] Tie Y., Huang Y., Chen R., Li L., Chen M., Zhang S. (2023). Current insights on the roles of gut microbiota in inflammatory bowel disease-associated extra-intestinal manifestations: Pathophysiology and therapeutic targets. Gut Microbes.

